# Immunogenicity of a Promiscuous T Cell Epitope Peptide Based Conjugate Vaccine against Benzo[a]pyrene: Redirecting Antibodies to the Hapten

**DOI:** 10.1371/journal.pone.0038329

**Published:** 2012-05-30

**Authors:** Mario T. Schellenberger, Nathalie Grova, Sophie Farinelle, Stéphanie Willième, Dominique Revets, Claude P. Muller

**Affiliations:** Institute of Immunology, Centre de Recherche Public-Santé/National Public Health Laboratory, Luxembourg, Grand Duchy of Luxembourg; National University of Singapore, Singapore

## Abstract

The prototype polycyclic aromatic hydrocarbon benzo[a]pyrene (B[a]P) is an environmental pollutant and food contaminant of epidemiological importance. To protect against adverse effects of this ubiquitous carcinogen, we developed an immunoprophylactic strategy based on a B[a]P-protein conjugate vaccine to induce B[a]P specific antibodies (Grova et al., Vaccine. 2009;27:4142–51). Here, we investigated in mice the efficacy of B[a]P-peptide conjugates based on promiscuous T cell epitopes (TCE) into further improve this approach. We showed that B[a]P-peptide conjugates induced very different levels of hapten-specific antibodies with variable functional efficacy, depending on the carrier. In some cases peptide carriers induced a more efficient antibody response against B[a]P than tetanus toxoid as a protein carrier, with the capacity to sequester more B[a]P in the blood. Reducing the carrier size to a single TCE can dramatically shift the antibody bias from the carrier to the B[a]P. Conjugates based on the TCE FIGITEL induced the best anti-hapten response and no antibodies against the carrier peptide. Some peptide conjugates increased the selectivity of the antibodies for the activated metabolite 7,8-diol-B[a]P and B[a]P by one or two orders of magnitude. The antibody efficacy was also demonstrated in their ability to sequester B[a]P in the blood and modulate its faecal excretion (15–56%). We further showed that pre-existing immunity to the carrier from which the TCE was derived did not reduce the immunogenicity of the peptide conjugate. In conclusion, we showed that a vaccination against B[a]P using promiscuous TCEs of tetanus toxin as carriers is feasible even in case of a pre-existing immunity to the toxoid and that some TCE epitopes dramatically redirect the antibody response to the hapten. Further studies to demonstrate a long-term protection of an immunoprophylactic immunisation against B[a]P are warranted.

## Introduction

Benzo[a]pyrene (B[a]P) is a ubiquitous environmental pollutant and food contaminant belonging to the group of polycyclic aromatic hydrocarbons (PAH). B[a]P is produced during incomplete combustion of organic matter and emanates from natural and anthropogenic sources including industrial processes, cooking, barbequing and tobacco consumption [1]. Uptake in humans is mostly by inhalation of contaminated air, cigarette smoke and ingestion of contaminated food or water. As a consequence exposure to B[a]P by the general public is unavoidable.

**Figure 1:**
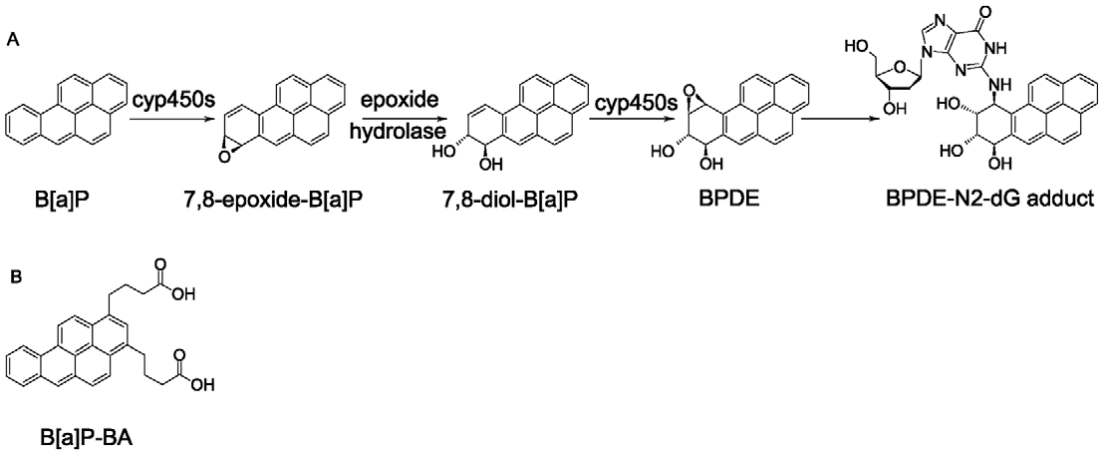
Metabolic activation of B[a]P. (A) During detoxification a small fraction of B[a]P is activated to 7-8-diol-B[a]P which is further converted to the highly reactive 7,8-dihydroxy-9,10-epoxy-B[a]P (BPDE) the ultimate DNA carcinogen. (B) Chemical structure of the Benzo[a]pyrene butyric acid isomeric mixture (B[a]P-BA), the derivative used for the conjugation to T cell epitope peptides.

Known adverse effects of B[a]P include carcinogenicity, immuno-, neuro-, geno-, reproductive and developmental toxicity [2]–[9]. B[a]P is a very effective pulmonary carcinogen in human and experimentally in rodents [10], [11]. The total dose experienced by a smoker in a lifetime is remarkably close to the lowest total dose shown to induce tumours in rats [12]. The aryl hydrocarbon receptor (AhR) plays an important role in B[a]P-induced carcinogenesis. Human and animal studies showed a significant correlation between the inducibility of the arylhydrocarbon hydroxylase activity and lung carcinogenesis induced by B[a]P [13], [14]. B[a]P mediated carcinogenicity can also be induced by its genotoxicity. Human lung and liver metabolically activate B[a]P to 7,8-diol-9,10-epoxide-B[a]P (BPDE) by phase one enzymes [15] (Figure 1). In human lung, DNA adducts of B[a]P have been detected [16], [17]. Metabolic manipulations by isothiocyanates that decrease the formation of DNA adducts, without lowering levels of chemical exposure, have been shown to reduce the number of tumours [16], [18]. Mechanistic studies have shown that the chemopreventive activity of isothiocyanates, that modify carcinogen metabolism specifically by inhibiting Phase one enzymes and/or by inducing Phase two enzymes, result in increased carcinogen excretion or detoxification and decreased carcinogen DNA interactions [19]. BPDE adducts have been linked to G:C to T:A transversions in the Tp53 gene at an unusual series of mutational hotspot codons in smoking-associated lung cancer [20]. Mutations in critical regions of this tumour suppressor gene or of oncogenes (e.g. Ras, Myc) can result in deregulation of normal cell growth and cancer development [21].

Therefore, we have started to develop strategies based on B[a]P-carrier conjugates to explore the ability of B[a]P specific antibodies to protect against the adverse effects of this carcinogen [22]–[27]. The use of hapten-carrier conjugates using proteins for vaccination have been successful in the case of nicotine and its major metabolite cotinine, or cocaine using various carrier proteins [28]–[31]. Some of these conjugates are already tested in clinical trials [29], [32]. However, only limited data are available for low molecular weight carcinogens [33]–[35]. Concerns about local carcinogenesis at the site of injection are probably unsubstantiated considering the low doses and the low metabolic activation rates of (conjugated) B[a]P in muscles in contrast to lung and liver tissues.

Our previous in vitro studies showed that monoclonal antibodies against B[a]P may provide some protection against low doses of B[a]P [22], [23]. In a bi-compartment model of polarised Caco-2 cells, we showed that antibodies reduced the transport of B[a]P and its metabolism by sequestration of B[a]P and its metabolites in the cell culture supernatant [22]. In addition, antibodies modulated the kinetic of B[a]P metabolism in HepG2 cells and human peripheral lymphocytes [23].

Our first in vivo experiments demonstrated that a hapten-protein conjugate vaccine based on tetanus toxoid (TT) and diphtheria toxoid (DT) in combination with various adjuvants licensed in humans was able to induce high levels of antibodies in mice which were specific for B[a]P, its detoxified metabolites and its activated form, the BPDE [25]–[27]. Although most experimental models use high concentrations of B[a]P to achieve an experimental read out after short-term exposure, such high concentrations cannot be used to study protection of B[a]P specific antibodies because they cannot be matched by stoichiometric concentrations of specific antibodies. However, environmental relevant concentrations of B[a]P can easily be matched by the antibody levels obtained by immunisation with B[a]P-TT conjugates [26] but they do not provide a short-term readout in relation to their carcinogenicity.

Conjugating the B[a]P to a bulky carrier protein (e.g. TT or DT) tends to divert the immune response to the immunogenic carrier protein and away from the hapten [36]. Carrier proteins have been replaced by small immunogenic peptides to induce prophylactic or therapeutic T cell responses against pathogens, tumours and other diseases but not against low molecular weight compounds such as B[a]P. In this study, we aimed to further investigate our prophylactic immunisation strategy against B[a]P using T cell epitope (TCE) peptides to redirect the antibody response to the hapten. TCEs were selected that were promiscuous with respect to a large variety of human and mouse MHC class II molecules.

## Materials and Methods

### 2.1. Peptide synthesis and B[a]P conjugation

Peptides (Table 1) were synthesised by automated solid phase peptide synthesis using standard Fmoc chemistry on Rink resin on a Syro II peptide synthesiser (Multisyntech, Witten, Germany). B[a]P butyric acid (B[a]P-BA, Biochemical Institute of Environmental Carcinogens, Grosshansdorf, Germany, Figure 1B) was coupled N-terminally to the protected peptide and was washed 3 times each with methanol and ether.

Peptide-B[a]P conjugates were purified by RP-HPLC on the ÄKTA explorer 10S system (Amersham Biosciences, Uppsala, Sweden) on a C18 column (250×8 mm, 120A, 5 µm) using a linear gradient of 25–95% water/acetonitrile (ACN), 0.1% TFA (v/v) and monitored at 214 nm (max absorption of peptide bond, [37]) and 297 nm (max absorption of B[a]P-BA) and lyophilized in an Alpha 24 lyophilisator (Christ, Osterode am Harz, Germany).

B[a]P-BA was coupled to ovalbumin (OVA, Sigma-Aldrich, Bornem, Belgium) and purified tetanus toxoid (TT, Serum Institute of India) by adopted two-step zero-length cross-linking procedure using active esters as described in Grova *et al*. [25].

**Table 1:** Summery of peptides and their expected and measured masses (mutations in the peptide sequence are in bold).

ID	Origin/Antigen/ [Ref]	Position	Sequence	Expected masses [M+H]^+^	Measured masses [M+H]^+^	Δ	Purity
VNNESSE–3	TT (wt–short) [41]	916–932	B[a]P–PGINGKAIHLVNNESSE	2098.100	2098.278	0.178	70%
VNNESSE–14	TT (wt–long)	909–932	B[a]P–PDAQLVPGINGKAIHLVNNESSE	2721.428	2721.513	0.085	95%
VNNESSE–15	TT		B[a]P–PDAQLVVGINGKAIHLVNNESSE	2723.443	2723.979	0.536	80%
VNNESSE–18	TT		B[a]P–AENKPGINGKAIHLVNNESSE	2540.317	2539.942	0.375	40%
VNNESSE–23	TT		B[a]P–ALAYYVLPGINGKAIHLVNNESSE	2891.537	2891.286	0.251	85%
VNNESSE–24	TT		B[a]P–PILFFRLKGINGKAIHLVNNESSE	3015.685	3015.178	0.507	80%
PNRDIL–8	TT (wt–short) [43]	1273–1284	B[a]P–GQIGNDPNRDIL	1630.862	1630.836	0.026	95%
PNRDIL–10	TT		B[a]P–ALGLVGTHNGQIGNDPNRDIL	2493.328	2493.416	0.088	80%
FIGITEL–6	TT (wt–short) [43]	830–844	B[a]P–QYIKANSKFIGITEL	2044.155	2043.896	0.259	90%
FIGITEL–29	TT (wt–medium)	826–844	B[a]P–NILMQYIKANSKFIGITEL	2515.406	2515.220	0.186	40%
FIGITEL–16	TT (wt–long)	823–844	B[a]P–QSKNILMQYIKANSK§ITEL	2858.592	2858.986	0.394	80%
FIGITEL–17	TT		B[a]P–QSKQILMVYIKANSKFIGITEL	2843.617	2843.606	0.011	90%
FIGITEL–21	TT		B[a]P–AENKQYIKANSKFIGITEL	2486.372	2486.182	0.190	40%
FIGITEL–27	TT		B[a]P–ALAYYVLQYIKANSKFIGITEL	2837.952	2837.872	0.080	60%
FIGITEL–28	TT		B[a]P–PILFFRLKYIKANSKFIGITEL	2930.734	2930.340	0.394	80%
SYFPSV–19	TT (wt) [41]	580–599	B[a]P–NSVDDALINSTKIYSYFPSV	2552.299	2552.572	0.273	90%
SYFPSV–20	TT		B[a]P–NSVDDALI**V**STKIYSYFPSV	2537.324	2537.514	0.190	95%
SYFPSV–22	TT		B[a]P–AENKALINSTKIYSYFPSV	2464.319	2464.154	0.165	70%

Tetanus toxoid (TT), wild type (wt).

### 2.2. Mass Spectroscopy

Masses of B[a]P-protein and -peptide conjugates were analysed using a positive ion MALDI-TOF ULTRAFLEX TOF/TOF mass spectrometry (Bruker Daltonics, Bremen, Germany) equipped with a 337 nm, 50 Hz N_2_ laser of 100 μj as described in [38]. For synthetic peptides the sequence was verified with the MALDI mass spectrometer using its post-source decay (PSD) capacity for fragmentation (Sequence Editor of Biotools software, Bruker Daltonics, Bremen, Germany). The N-terminal linkage of B[a]P on peptides was determined by MS/MS using an ion trap mass spectrometer (Agilent 6340 ion trap) equipped with a Chip Cube interface for infusion at nano flow rate. The parameter settings for positive ion ESI-MS were as follows: capillary voltage 2000 V; end plate offset 500 V; capillary exit 100 V and tarp drive 85. For CID, the fragmentation amplitude was set to 1.3 V scanned from 30% to 200% of this value.

### 2.3. Immunisation with B[a]P-peptide conjugates

All animal experiments were done in compliance with the rules of the European Communities Council Directive of 24 November 1986 (86/609/EEC) and were approved by the Ministry of Agriculture, Viticulture and Rural Development (22 December 2008). A group of 6 mice Balb/c (10 week olds, female, Harlan, Horst, The Netherlands) were immunised as described previously [26]. Briefly, mice were primed i.p. with 25 μg of B[a]P-peptide or B[a]P-TT bioconjugates (50 mM ammonium bicarbonate buffer) on day 0, emulsified in 50% complete Freund's adjuvant (CFA, v/v in PBS, Sigma-Aldrich). On days 14, 28 and 42 mice were boosted i.p. with the same antigen emulsified in 50% incomplete Freund's adjuvant (IFA, v/v in PBS, Sigma–Aldrich,). Mock immunised control group (n = 5) was primed with 50% CFA and boosted with 50% IFA (v/v in PBS) alone.

### 2.4. Detection of specific antibodies after immunisation

**Figure 2:**
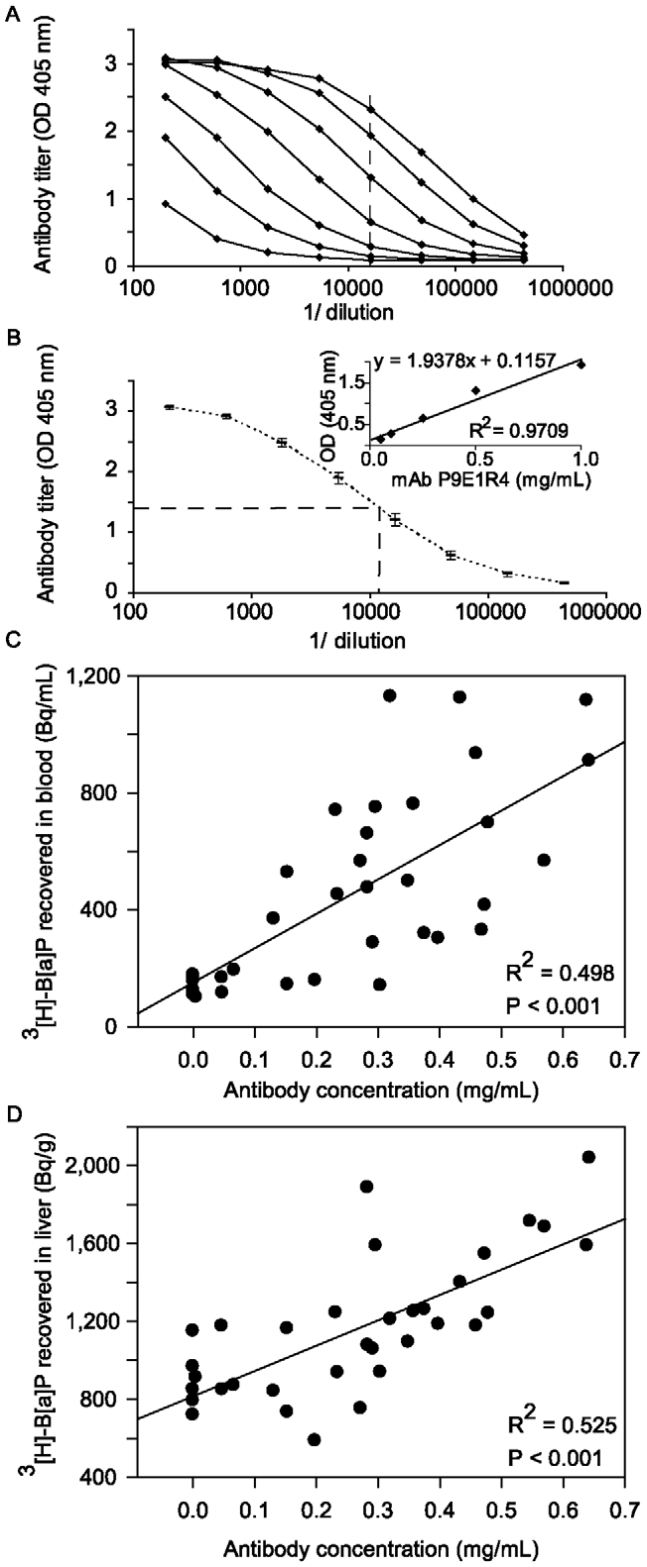
Anti-B[a]P antibody concentration determined by indirect ELISA and correlation with antibody levels. To estimate absolute antibody concentrations a standard curve (insert in panel B) was used, that was based on dilution curves (1/200–1/437,400) of different known concentrations (0.01–1 mg/ml, represented by a line for each concentration in panel A) of purified monoclonal antibody against B[a]P (P9E1R4). 1/16,200 dilution (dashed line) was chosen to plot the standard curve (Insert in panel B, R^2^ = 0.9709) and to calculate the antibody concentration. (B) Example of a titration curve of serum antibodies of mice immunised with B[a]P conjugated TT 2 weeks after the fourth injection. Values are presented as mean ± S.E.M of 6 mice per group. (C, D) Correlation between antibody level and [^3^H]-B[a]P recovery after a single i.p. injection of 2 μg/kg [^3^H]-B[a]P in individual mice mock immunised and immunised against B[a]P using B[a]P-peptide or B[a]P-TT conjugates. [^3^H]-B[a]P recovery in the blood (C) and liver (D). The statistical significant relationship between the two variables as shown in C and D was estimated by linear regression.

On day 53, mice were bled retro-orbitally and serum antibody levels were determined in 384-well microtiter plates (Greiner, Wemmel, Belgium) as described previously [26]. Briefly, microtiter plates were coated overnight at 4°C with 0.25 μM B[a]P-OVA (100 mM carbonate buffer, pH 9.6) for detection of B[a]P specific antibodies or with homologous non conjugated peptides (VNNESSE–14, –15, FIGITEL–16, –17 or TT) for the quantification of carrier specific antibodies. After washing, free binding sites were saturated with 1% BSA at RT for 2 h. After washing, diluted serum or carcinogen-specific mouse monoclonal antibody (P9E1R4 produced by immunisation with B[a]P–DT [25]) was incubated for 90 min at RT. Binding was assessed by alkaline phosphatase-conjugated goat anti-mouse IgG (1/750 dilution, ImTec Diagnostics NV, Antwerpen, Belgium) and 4-nitrophenyl phosphate disodium salt hexahydrate (Sigma-Aldrich). Absorbance was measured at 405 nm (Spectromax Plus, Sopachem, Brussels, Belgium). For relative quantification, endpoint titers (EPT) were defined as serum dilutions corresponding to 5 fold the background. Absolute antibody quantification is described in [26] and Figure 2A and B. For sera with no detectable antibodies, endpoint titer was set to 200 (highest serum concentration tested).

### 2.5. Antibody selectivity by competition ELISA

The selectivity of serum antibodies was determined by competition ELISA as described previously [39]. Briefly, B[a]P-BA, B[a]P (Sigma–Aldrich) and 7,8-diol-B[a]P (National Cancer Institute Chemical Carcinogen Reference Standard Repository, Midwest Research Institute, Kansas City, MO, USA) were used as competitors to inhibit antibody binding to coated heterologous B[a]P-conjugate (B[a]P–OVA). For these competition experiments, the optimal amount of coating antigen and the serum dilution were determined by indirect ELISA as described above. The minimal amount of antigen required for saturation was coated on 384-well microtiter plates. Sera dilutions were determined to obtain 70% of saturation. Dilution series of the competitors were mixed 1∶1 with the diluted serum to final concentrations of 0 to 1024 μM. No competition (highest signal) and 100% competition (background signal) were determined using no competitor. The difference between the two values corresponds to the dynamic range of the assay (Δ OD max). For each competitor concentration, the percent binding of antibody was determined using the following formula:

% binding  =  [(OD_- test_ *–* OD_+ background_)/Δ OD max *100].

From the resulting inhibition curves, the 50% inhibition concentration (IC_50_) of each competitor was determined. In mice without selectivity, the IC_50_ were actively set to 1 mM. A low IC_50_ value corresponds to high selectivity.

### 2.6. Distribution of [^3^H]-B[a]P in immunized mice after a single injection

The quantification of [^3^H]-B[a]P in organs and excretion products were described before [25]. Briefly, two weeks after a complete immunisation schedule, each animal received a single i.p. injection of [^3^H]-B[a]P (2 μg/kg, 2.67 10^4^ Bq/mouse GE-Healthcare, Belgium) and were placed in individual metabolic cages (Technilab, Someren, Netherlands). Urine, faeces and organs (Liver, Lung, Brain, Spleen, and Kidney) were collected 24 h later and stored at – 20°C before analysis. EDTA-blood (500 µL) was obtained by retro-orbital bleeding and analysed immediately. To study the pharmacokinetic of B[a]P, mice were challenged with 2 μg/kg [^3^H]-B[a]P (2.67 10^4^ Bq/mouse) and sacrificed after 15 min to 48 h intervals. Samples were collected and stored as described above.

Samples were analysed for [^3^H]-B[a]P recovery as described previously [39]. Pre-hydrated faeces (20 mg) or tissues (100 mg) were solubilised in Soluene-350 according to Perkin Elmer procedure and previously described and measured for radioactivity [40]. All samples were corrected for quenching of radioactivity. Radioactivity was expressed in Bq or Bq/g accounting for the effective counting efficiency.

## Results

### 3.1. T cell epitope peptide synthesis and B[a]P conjugation

**Figure 3:**
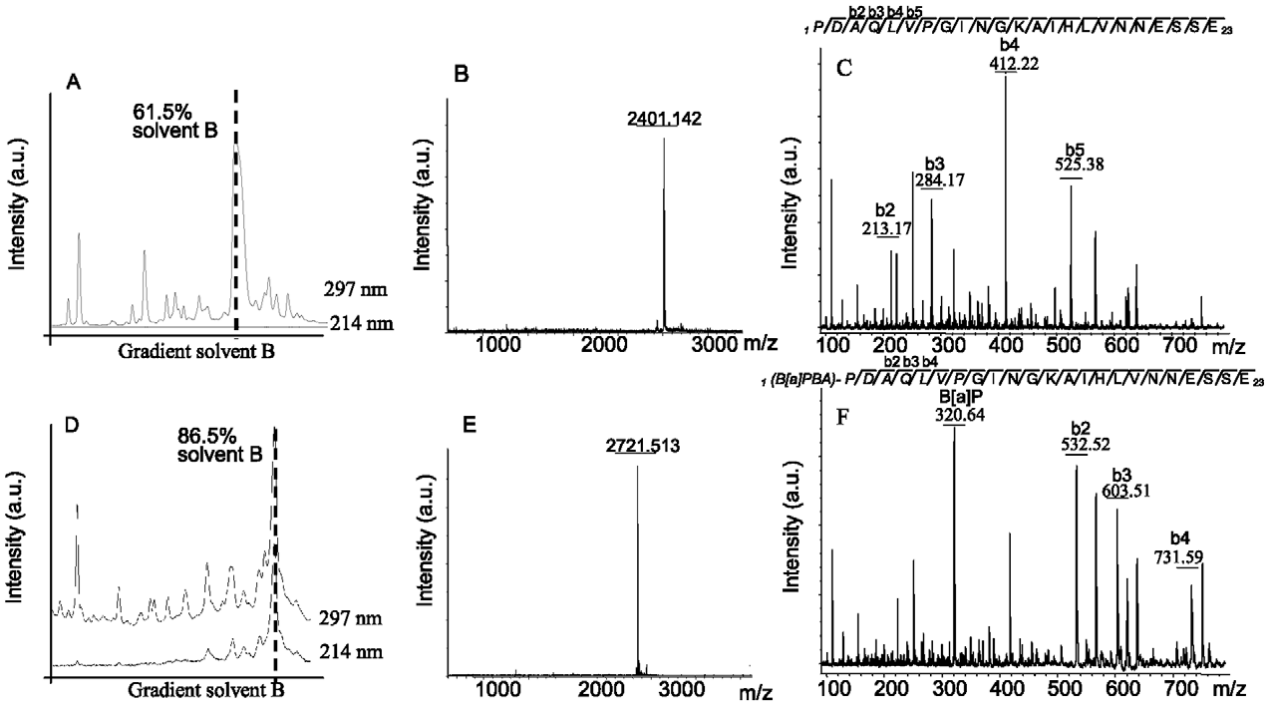
Peptide conjugation. HPLC chromatogram before purification (A, D). MS/MS spectrum (B, E) and the N terminal sequence (C, D) of VNNESSE-14 after purification. Panel A, B, C represent the unconjugated and panel D, E and F the B[a]P-BA conjugated peptide.

After solid phase synthesis peptides were purified by liquid chromatography and analysed by positive ion MALDI-TOF ULTRAFLEX TOF/TOF mass spectrometry for their correct masses. All synthesised peptides had the correct molecular mass (Table 1). The Figure 3B shows as an example the MS spectrum of peptide VNNESSE-14. After conjugation, the mass spectra showed the correct shift in size of 320 Da corresponding to B[a]P-BA (Figure 3B and E). In addition, a shift in the elution from the HPLC from 61.5% to 86.5% ACN confirmed the successful conjugation of B[a]P-BA to the peptide by its increased hydrophobicity (Figure 3A and D).

The peptide sequence and the N-terminal linkage of the B[a]P-BA were also confirmed by MS/MS analysis. The peak of 320.64 Da corresponds to the [B[a]P-BA+H]^+^ molecule (Figure 2F). The mass difference of 320.64 Da (corresponding to the [B[a]P-BA+H]^+^) between the b2 ions in Figure 3F and C proves the N-terminal linkage.

### 3.2. Detection of B[a]P- specific antibodies after immunisation with B[a]P-peptides

**Figure 4:**
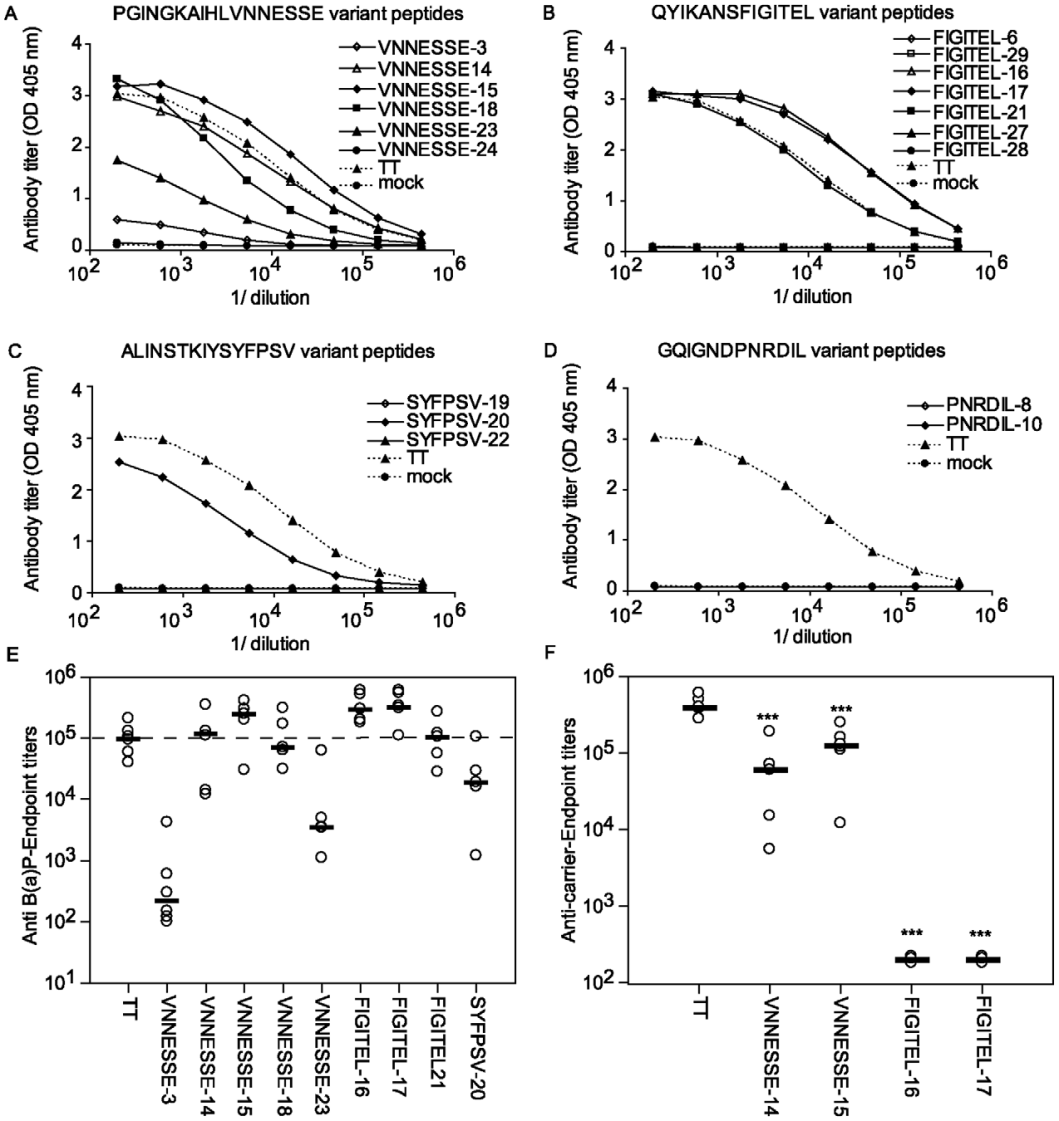
Antibody titration curves of sera from mice immunised with B[a]P-peptide conjugates. (A) VNNESSE-variants, (B) FIGITEL-variants, (C) SYFPSV-variants and (D) PNRDIL-variants. Control mice were immunised with B[a]P conjugated to TT-protein (dashed line). Values are mean of 6 mice per group determined by indirect ELISA using heterologous conjugates (B[a]P-ovalbumin) as coated antigen. (E) Serum endpoint titers (serum dilution reaching 5 times the background) of B[a]P specific IgG antibodies of individual mice (○) and median value (–). Groups correspond to a selection of panel A to D. Dashed line represents the endpoint titer for immunisation with tetanus toxoid. (F) Endpoint titers against homologous carrier peptide. For sera with no detectable antibodies endpoint titers were set to 1/200. Results are presented for each mouse (○) and median value (–). ***p<0.001, statistically significant difference from TT immunised mice (One Way ANOVA test followed by Student-Newman-Keuls t-test for multiple comparison).

Four known TCE of different length of TT were conjugated to B[a]P-BA (VNNESSE-3, -14, FIGITEL-6, -16, -29, PNRDIL-8 and SYFPSV-19, Table 1) and tested for their potential to induce B[a]P specific antibodies. These peptides reacted with mouse H2^d^ and were promiscuous for human MHC class II molecules [41]–[44]. For instance according to the literature FIGITEL peptides react with most DR and some DQ and DP MHC class II molecules whereas PNDRL and SYFPSV peptides react with a variety of human DR molecules and some mouse class II molecules [42], [44]. In addition, B[a]P was conjugated to several of variants of these TCE peptides. The serum of each mouse was titrated 2 weeks after the fourth injection. Figure 4 shows the variable immunogenicity of a selection of B[a]P-peptide conjugates. From B[a]P conjugates based on VNNESSE-motive only VNNESSE-14 and -15 (EPT 1/120,000 and 1/250,000) induced higher levels of specific antibodies against B[a]P (Figure 4A and E) than the B[a]P-TT protein conjugate (EPT 1/99,500, Figure 4E) but EPT did not significantly differ. All other VNNESSE-peptides induced significantly lower levels or no antibodies (Figure 4A and E). Peptide conjugates from the FIGITEL group which induced higher levels of B[a]P specific antibodies than B[a]P-TT included FIGITEL-16 and -17 (EPT 1/300,000 and 1/325,000 respectively, Figure 4B and E). Only peptide SYFPSV-20 (EPT 1/19,000, Figure 4C and E) induced specific antibodies in the group of SYFPSV peptides, but less efficient than B[a]P-TT. None of the tested peptides of the PNRDIL-motive induced specific B[a]P antibodies (Figure 4D).

### 3.3. Detection of carrier specific antibodies after immunisation with B[a]P-peptides

The sera with the highest titers against B[a]P were further tested for antibodies against the homologous carrier peptide. Mean antibody levels were 6.5 times lower for VENNESSE-14 (EPT 1/60,000) and 3 times lower for VENNESSE-15 (EPT 1/125,000) compared to immunisation with B[a]P-TT (EPT 1/390,000 Figure 4F). Interestingly for the FIGITEL-16 and -17 conjugates no antibodies against the carrier were detected (EPT <1/200).

### 3.4. Antibody selectivity by competitive ELISA

**Figure 5:**
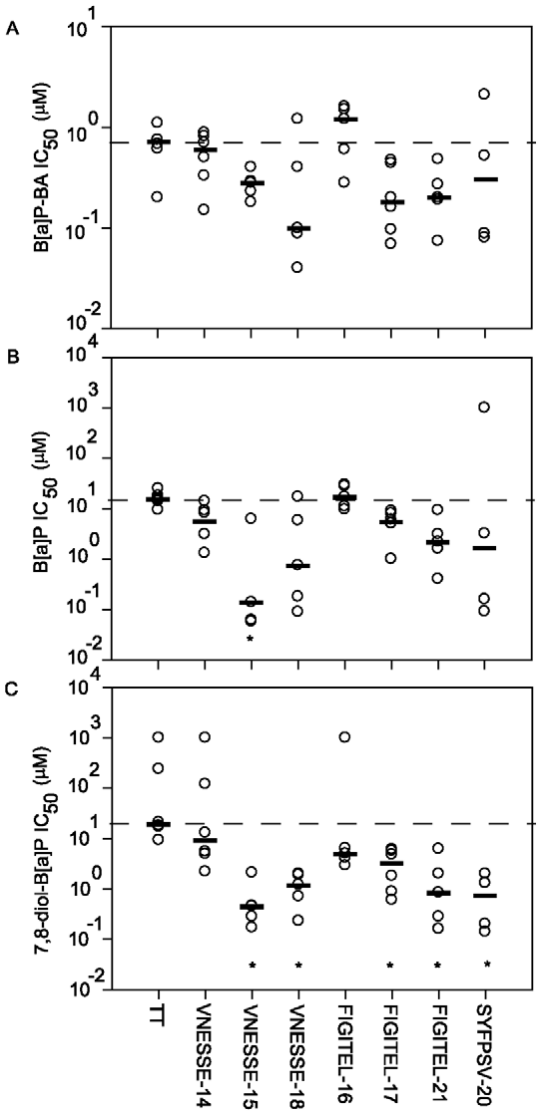
Antibody selectivity determined by competitive ELISA in sera immunised with B[a]P peptide or TT conjugates. (A) B[a]P-BA, (B) B[a]P, and (C) 7,8-diol-B[a]P were used as competitors to compete for binding of specific antibodies to B[a]P-ovalbumin as the coated antigen. The IC_50_ (concentration of competitor for 50% inhibition) was calculated as a measure of antibody selectivity for each tested competitor. The IC_50_ is inversely correlated to the antibody selectivity. Results are represented for each mouse (○) and median value (–).*p<0.05, statistically significant difference from TT immunised mice (One Way ANOVA on Ranks followed by Dunn's method).

The selectivity of the anti-B[a]P antibodies was analysed by competitive ELISA in all sera of mice with detectable antibodies against B[a]P. In a competitive ELISA assay IC_50_ values inversely correlate with the selectivity for the competitor. IC_50_ values for B[a]P-BA as competitor ranged from 0.7 µM for B[a]P-peptide conjugate VNNESSE-18 to 1.20 µM for FIGITEL-16 (Figure 5A). Compared to B[a]P-TT, the antibodies induced by VNNESSE-15, -18, FIGITEL-17, -21 and SYFPSV-20 showed a somewhat higher selectivity for B[a]P-BA (although not statistically significant). Competition with B[a]P, gave IC_50_ values that were 5 to 30 times higher than those of B[a]P-BA (except for VNNESSE-16 with similar IC_50_ values) ranging from 0.14 µM for B[a]P-peptide conjugate VNESSE-15 to 17.00 µM for FIGITEL-16 (Figure 5B). The IC_50_ of 7,8-diol-B[a]P was also higher than those of B[a]P-BA ranging from 0.45 µM (VNESSE-15) to 9.25 µM (VNESSE-14, Figure 5C). All tested peptide conjugates, except the FIGITEL-16, showed also a higher selectivity for B[a]P and 7,8-diol-B[a]P than the TT protein conjugate.

### 3.5. Modulation of [^3^H]-B[a]P distribution in immunised mice 24 h after a single injection

**Figure 6:**
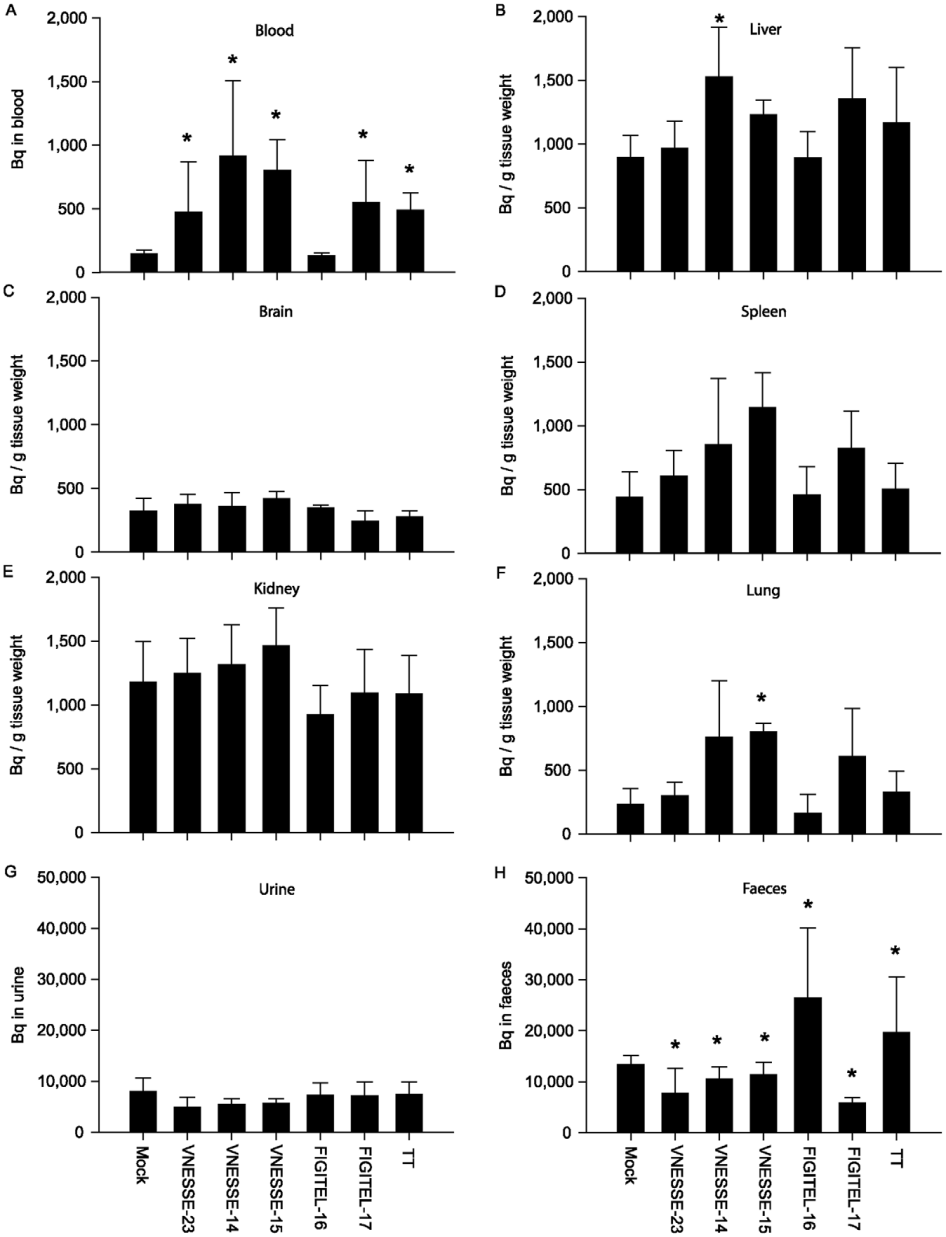
B[a]P recovery in mice immunised against B[a]P. [^3^H]-B[a]P (in Bq/g tissue weight or Bq) recovered in blood (A), tissues (B–liver, C–brain, D–spleen, E–kidney, F–lung), urine (G) and faeces (H) 24 h after a single i.p. injection of [^3^H]-B[a]P (2 μg/kg) in B[a]P-peptide, B[a]P-TT or mock immunised mice. Results are expressed as mean ± S.E.M of 5 mice per group. *p<0.05; statistical significant difference from control (Mock) (Student-Newman-Keuls-t test for multiple comparisons).

[^3^H]-B[a]P recovery in the blood was on average 3-6 times higher than in mock immunised animals (p<0.05, Figure 6A) for all peptide conjugates tested except for FIGITEL-16. VNNESSE-14 and -15 increased [^3^H]-B[a]P recovery above the level of B[a]P-TT immunised animals but the difference was not statistically significant (Figure 6A). In the different solid tissues tested [^3^H]-B[a]P recovery was on average 1.1-2.6 fold increased (Figure 6B–F). The difference compared to the mock immunised animals was highest in the liver (Figure 6B) and the spleen (Figure 6D) for VNNESSE-14 and VNNESSE-15 (but this did not reach statistical significance) respectively. No difference was observed in the brain (Figure 6C). Animals immunised with FIGITEL-16 showed no enhanced retention of [^3^H]-B[a]P in any of the four organs tested. In faeces, a significant decrease of 15–56% of the radioactivity recovered was observed (p<0.05) in mice immunised with peptide conjugates (except for FIGITEL–16) while no decrease was observed for B[a]P-TT (Figure 6H). Excretion of radioactive B[a]P in the urine was essentially the same in both immunised and mock control mice (Figure 6G). Fig 5 shows that there is a good and highly significant correlation between increasing B[a]P antibody concentration and levels of [^3^H]-B[a]P recovered in the blood and in the liver (Figure 2C and D).

### 3.6. Modulation of the pharmacokinetic of [^3^H] -B[a]P

**Figure 7:**
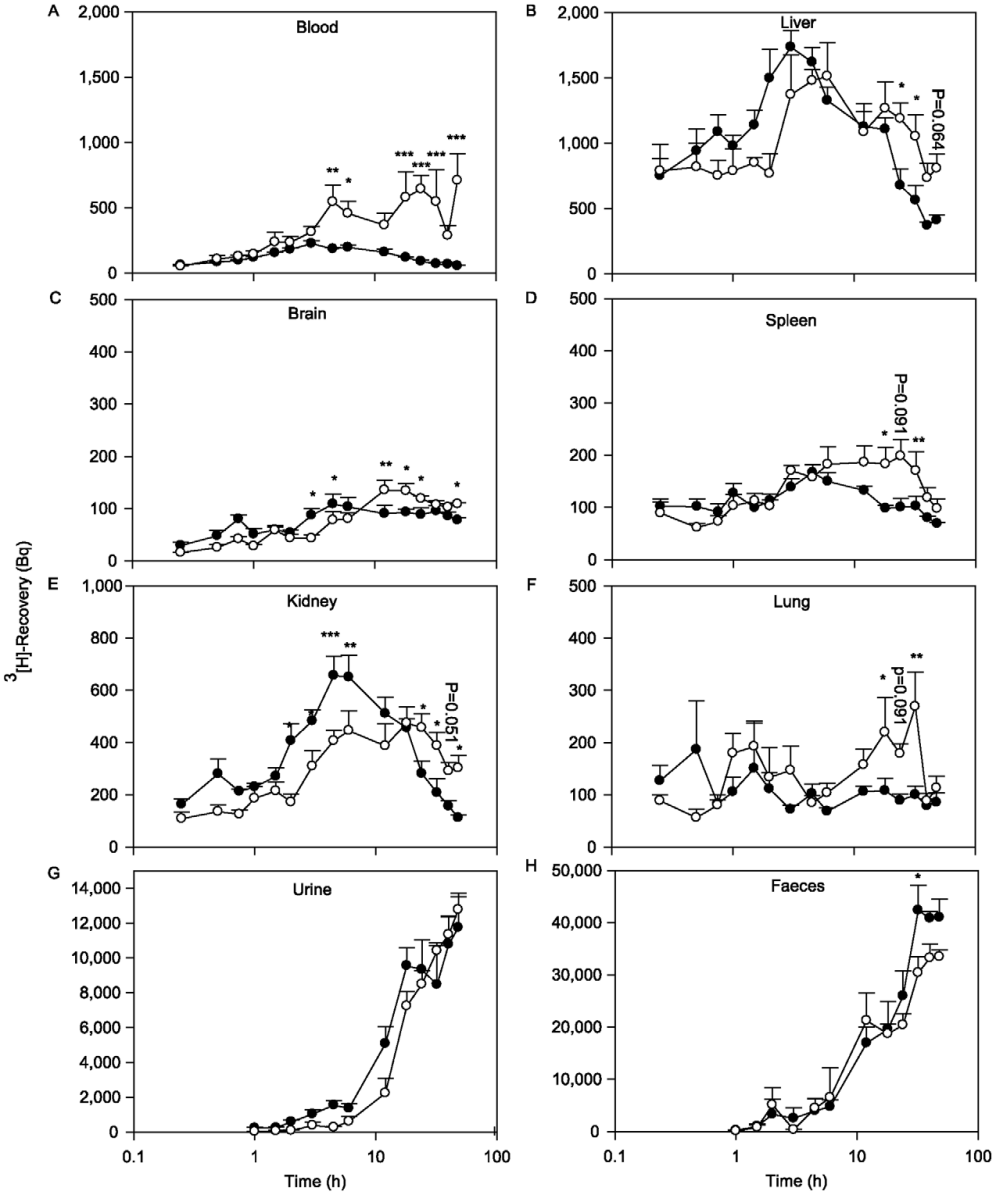
B[a]P pharmacokinetic. Pharmacokinetic of [^3^H]-B[a]P in mice immunised with VNESSE-14 (○) and mock immunised mice (○) over 48 hours after a single i.p. injection of 2 μg/kg [^3^H]-B[a]P. (A) blood, (B) liver, (C) brain, (D) spleen, (E) kidney, (F) lung, (G) urine, (H) faeces. Results are expressed as mean ± S.E.M of 5 mice per group. *p<0.05; **p<0.01 and ***p<0.001, statistical significant difference from controls (Two way ANOVA procedure followed by Student-Newman-Keuls-t test).

The pharmacokinetic of B[a]P was investigated over 48 h in blood, solid tissues and excretion products in mice immunised with peptide VNNESSE-14 (Figure 7). In mock immunised mice a rapid accumulation of [^3^H]-B[a]P was observed in the blood with peak concentrations 3 h after B[a]P administration (Figure 7A). In B[a]P immunised mice a significantly higher peak was observed 4.5 h after B[a]P injection and was constant for at least 48 h (Figure 7A). In solid tissues, the highest levels of B[a]P were detected in the liver and the kidney, the lowest in the brain 3-4.5 h after injection. In B[a]P immunised mice, the peak concentration was delayed by 1-2 h. In kidney and brain, the concentration of B[a]P was lower in the immunised group during the uptake phase (first 10 h), and was then constant over the observation period, while a decrease was observed for the kidney in the control group (Figure 7C and E). For the other organs tested (liver, spleen, lung) no difference was observed early after B[a]P administration (Figure 7B, D and F). As a consequence of B[a]P sequestration by antibodies, excretion in the urine and faeces was reduced (Figure 7G and H). In the urine of immunised mice, lower levels of B[a]P were detected during the first 18 h, whereas in the faeces this difference was only observed during the first 3 h (Figure 7G and H).

### 3.7. Effect of a TT pre-vaccination on the B[a]P vaccination

**Figure 8:**
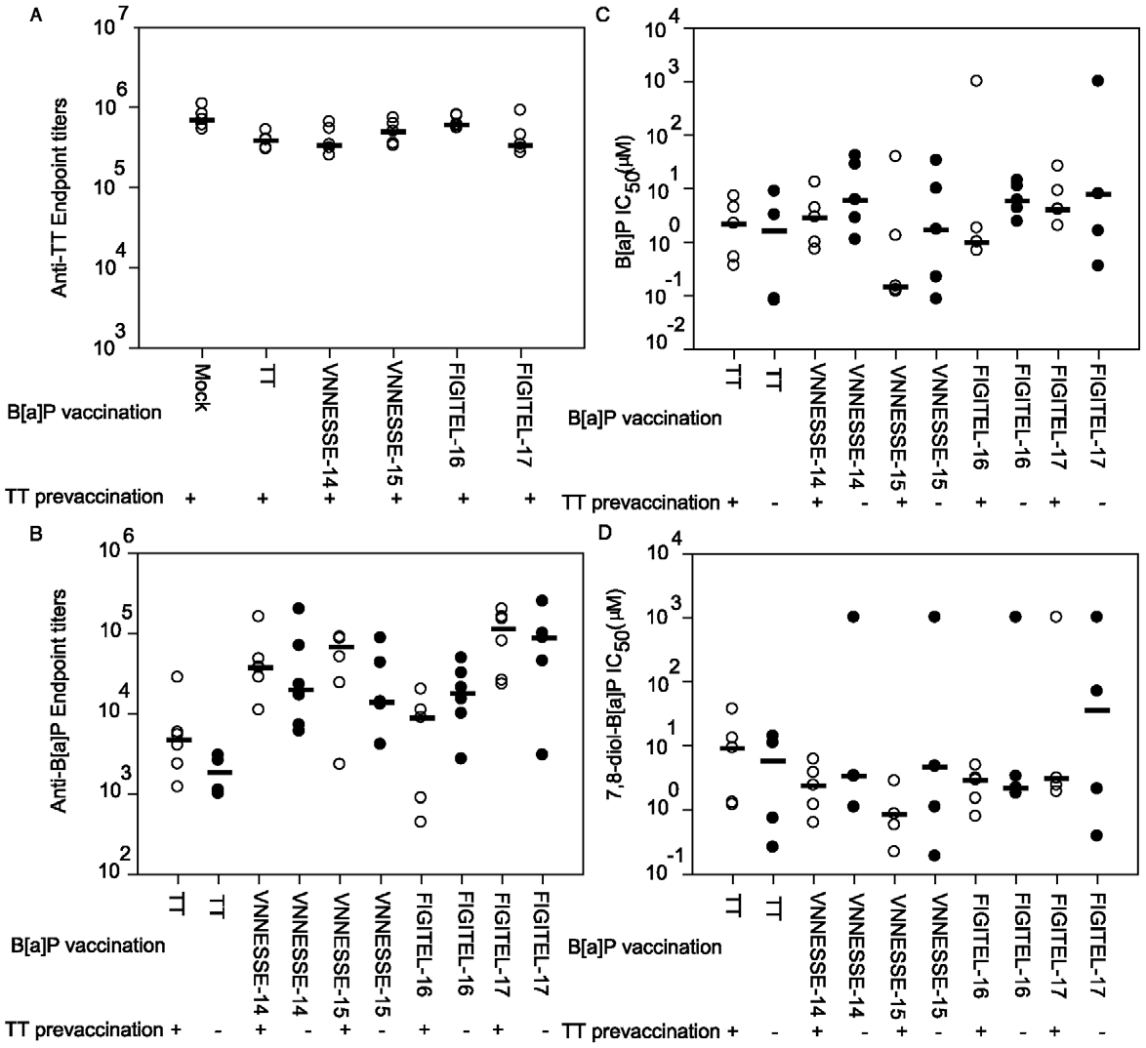
Immunogenicity of B[a]P-peptide conjugates after tetanus toxoid pre-vaccination. (A) Endpoint titers (serum dilution reaching 5 times the background) for TT specific IgG antibodies determined by indirect ELISA for sera pre-immunised with tetanus toxoid (TT) followed by B[a]P-peptide or B[a]P-TT conjugate vaccination. (B) B[a]P specific IgG antibodies with and without pre-vaccination. Results are presented for each mouse (○) and median value (–). There was no statistical significant difference between animal with and without pre-vaccination (One way ANOVA procedure followed by Student-Newman-Keuls-t test). (C, D) Antibody selectivity determined by competitive ELISA in sera immunised with B[a]P peptide or B[a]P-TT conjugates with (○) and without (•) tetanus toxoid (TT) pre-vaccination. B[a]P (C) and 7,8-diol-B[a]P (D) were used as competitors to compete for binding of specific antibodies to B[a]P-ovalbumin as the coated antigen. The IC_50_ (concentration of competitor for 50% inhibition) was calculated to determine the antibody specificity for each tested competitor. The IC_50_ is inverse correlated to the antibody affinity. Results are presented for each mouse (circle) and median value (–).

**Figure 9:**
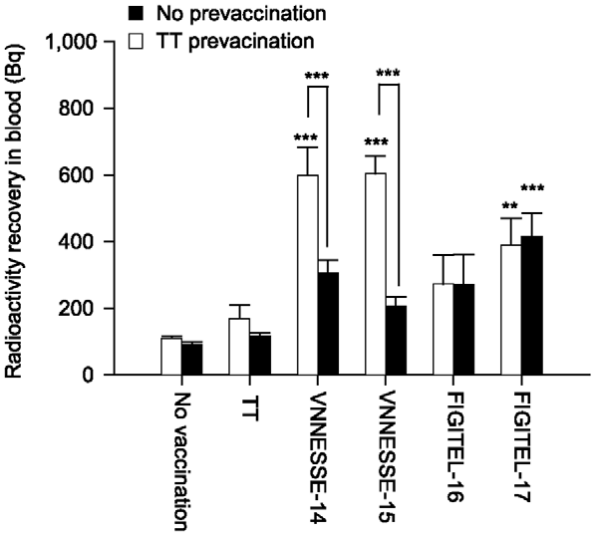
B[a]P recovery in mice vaccinated against B[a]P and tetanus toxoid. [^3^H]-B[a]P recovered in blood 24 h after a single i.p. injection of [^3^H]-B[a]P (2 µg/kg) in mice immunised with B[a]P-peptide or B[a]P-TT conjugates, with (open bars) or without (closed bars) tetanus toxoid (TT) pre-vaccination. Results are expressed as mean ± S.E.M of 5 mice per group. **p<0.01, ***p<0.001 significant difference from controls (Two Way ANOVA followed by Bonferoni). Control groups are No pre-vaccination (closed bars) or animals without B[a]P vaccination (No vaccination).

The influence of a pre-existing immune response to the carrier protein (TT) was tested by immunising mice with TT prior to immunisation with B[a]P-peptides and B[a]P-TT. Antibody levels in mice pre-immunised with TT were higher or similar to those without pre-vaccination, excluding a negative effect of pre-existing antibodies to TT (Figure 8B). Also the antibodies against TT were not influenced by a vaccination with B[a]P-peptide conjugates (Figure 8A). There was also no negative effect of TT pre-exposure on antibody specificity for B[a]P or 7,8-diol-B[a]P (Figure 8C and D). The in vivo recovery experiment also reflected this observation (Figure 9). 24 h after [^3^H]-B[a]P injection the recovery of radioactivity was similar for FIGITEL-16 and -17 compared to those without pre-vaccination and for VNNESSE-14 and -15 it was even significantly increased.

## Discussion

In our previous work we showed that the conjugation of B[a]P-BA to TT or diphtheria toxoid (DT) induces high titers of B[a]P-specific antibodies [25]. Here we demonstrate that similar or even better B[a]P-specific antibody titers can be induced by reducing the carrier size to a single promiscuous TCE.

It was previously shown that vaccination with a co-linear peptide containing a peptide corresponding to an important neutralising epitope of the measles hemaglutinin protein and various TCEs induced antibodies with activities that ranged from simply binding to in vitro neutralisation and in vivo protection against the virus [45]. Similarly, we here show that TCE-peptides induced very different levels of hapten specific antibodies with varying functional efficacies, depending on the carrier peptide. In some cases the peptide carrier induced a more efficient immune response than the protein conjugates. Not all known TCE-peptides [41]–[43] induced hapten specific antibodies (Figure 4A), which may reflect difficulties of the antigen processing and presentation machinery to properly cleave the conjugate.

Peptide conjugates are simple and cost effective to synthesise in large quantities, in high quality and safety [46]. In addition, peptide carrier conjugates are stoichiometrically well defined (Figure 3F) which is not the case of protein conjugates. The TT-protein carrier conjugate had on average 8 haptens per molecule, corresponding to 1 hapten for 18 kDa of protein [25], while the hapten/carrier ratio was 1 in the case of the peptide conjugate, corresponding to 1 hapten per 3 kDa of carrier. Under the simplest assumption that the molecular weight is a crude estimate of the relative number of B cell epitopes (BCEs), the peptide conjugate would have a six fold better ratio of hapten to carrier BCEs. Indeed, while some peptide conjugates induced similar or even higher anti B[a]P titers, antibody levels against the carrier peptide was up to 6 fold lower for some of the VNESSE conjugates. Interestingly, the conjugate FIGITEL-16 and -17 induced the best anti-hapten responses and no antibodies against the carrier peptide. Although this may be suggestive of a hole in the B cell repertoire despite a strong T cell immunogenicity against the latter peptide, vaccine conjugate based on appropriate small peptide carriers strengthen the B cell response towards the hapten at the detriment of the carrier.

Some peptide conjugates did not only induce similar or higher levels of antibodies against B[a]P, but they also showed an increased specificity and an improved ability to sequester [^3^H]-B[a]P. In particular peptide VNESSE-14 and -15 showed an improved immune response in terms of antibody quantity, quality and B[a]P sequestration in the blood.

Our TCE were derived from TT, against which 90% of the world population is vaccinated. Thus a large proportion of a population will have T cells specific for the promiscuous TCE selected here. To exclude that a pre-existing T cell immunity against TT interfere with the immune response against the TCE (from TT) undermining the anti-hapten antibody response, we tested the effect of a TT pre-vaccination on the vaccination with B[a]P peptide-conjugates. B[a]P antibody levels were in general higher in pre-immunised animals for all peptide conjugates tested (except for FIGITEL-16, Figure 8). Also antibody specificity and effectiveness did not suffer as a result of pre-vaccination with TT, for most of the peptide conjugates. In fact, the recovery of [^3^H]-B[a]P was significantly increased in mice pre-immunised with TT and boosted with VNESSE-14 and -15. These results are in agreement with Putz et al. who tested the immunogenicity of B cell epitope peptides conjugated to DT or TT derived TCE in mice after active priming with the toxoids [47]. Both TT and DT peptide conjugates induced high titers of anti-measles antibodies which cross-reacted with the virus and protected against a lethal challenge with the virus, even after active priming with the homologous toxoid [47]. Similar observations were found in humans, active priming against the carrier enhanced the response to the antigen conjugated to TT and DT irrespective of whether proteins, or peptides were used [48]–[51].

Kinetic experiments showed that B[a]P specific antibodies modulate the pharmacokinetics and slow down its excretion by capturing B[a]P or its metabolites in the blood stream and by reducing its excretion via the faeces (Figure 2H). The higher levels of B[a]P recovered in solid tissues in immunised mice are due to sequestration by antibodies and not by the blood in the organs. The results are in accordance with Johanson and colleagues who showed a higher recovery of dinitrophenol in the liver (20% in spleen and 50% lung) in rats passively immunised with anti-dinitrophenol antibodies [52]. B[a]P specific antibodies are able to capture B[a]P or its metabolites in the blood away from sensitive tissues and thereby mitigating P450 enzyme induction and reducing its metabolism especially to its toxic metabolite 7,8-diol-9,10-epoxide-B[a]P. In general antigen-antibody complexes are depleted mostly by Kupffer cells of the reticuloendothelial system of the liver [52]–[54]. Because of the lower sensitivity of the liver to B[a]P carcinogenesis, metabolism of B[a]P in this organ is much less likely to cause damage. In addition, we can speculate that the sequestration by specific antibodies of environmental (i.e. very low) concentrations of B[a]P (mean daily uptake 200 ng [55]) would be considerably higher resulting in an even better protective effect against its toxicity.

In previous experiments we demonstrated in vitro and in vivo that B[a]P specific antibodies are also able to capture its endpoint metabolites and the 7,8-diol which is the precursor of the ultimate carcinogen 7,8-diol-9,10-epoxide-B[a]P [23], [26]. In addition, our kinetic data show that B[a]P and its metabolites are captured for a prolonged time in the blood (Figure 7A). This lowers intracellular peak concentrations preventing enzyme induction of Cyp1a1 and 1b1 responsible for the formation of 7,8,-diol-9,10-epoxide-B[a]P [26].

In conclusion, we demonstrated that a vaccination against B[a]P using promiscuous T-helper cell epitopes as carriers is feasible. Some peptide conjugates were more immunogenic and induced more and better antibodies against the hapten. We further showed that appropriate small peptide carriers can redirect the antibody response against the hapten at the detriment of the B cell response to the carrier. This effect may partially explain the improved response to these peptide conjugates. Pre-exposure to TT did not negatively affect the immune response against B[a]P-peptide or B[a]P-TT conjugates. This lends further support to the use of TT derived peptides or protein as carriers for an immunoprophylactic conjugate vaccine against low molecular weight carcinogens such as B[a]P. While we demonstrated previously that our vaccination strategy is protective against short term adverse effects of B[a]P, such as immunotoxicity [26] and neurotoxicity (unpublished data), further studies to demonstrate a long term protection against carcinogenesis are needed.
